# SAMbinder: A Web Server for Predicting S-Adenosyl-L-Methionine Binding Residues of a Protein From Its Amino Acid Sequence

**DOI:** 10.3389/fphar.2019.01690

**Published:** 2020-01-30

**Authors:** Piyush Agrawal, Gaurav Mishra, Gajendra P. S. Raghava

**Affiliations:** ^1^ Department of Computational Biology, Indraprastha Institute of Information Technology, New Delhi, India; ^2^ Bioinformatics Center, CSIR-Institute of Microbial Technology, Chandigarh, India; ^3^ Department of Electrical Engineering, Shiv Nadar University, Greater Noida, India

**Keywords:** S-adenosine-L-methionine, PSSM profile, *in silico* prediction, cancer, machine learning technique (MLT)

## Abstract

**Motivation:**

S-adenosyl-L-methionine (SAM) is an essential cofactor present in the biological system and plays a key role in many diseases. There is a need to develop a method for predicting SAM binding sites in a protein for designing drugs against SAM associated disease. To the best of our knowledge, there is no method that can predict the binding site of SAM in a given protein sequence.

**Result:**

This manuscript describes a method SAMbinder, developed for predicting SAM interacting residue in a protein from its primary sequence. All models were trained, tested, and evaluated on 145 SAM binding protein chains where no two chains have more than 40% sequence similarity. Firstly, models were developed using different machine learning techniques on a balanced data set containing 2,188 SAM interacting and an equal number of non-interacting residues. Our random forest based model developed using binary profile feature got maximum Matthews Correlation Coefficient (MCC) 0.42 with area under receiver operating characteristics (AUROC) 0.79 on the validation data set. The performance of our models improved significantly from MCC 0.42 to 0.61, when evolutionary information in the form of the position-specific scoring matrix (PSSM) profile is used as a feature. We also developed models on a realistic data set containing 2,188 SAM interacting and 40,029 non-interacting residues and got maximum MCC 0.61 with AUROC of 0.89. In order to evaluate the performance of our models, we used internal as well as external cross-validation technique.

**Availability and Implementation:**

https://webs.iiitd.edu.in/raghava/sambinder/.

## Introduction

Structural and functional annotation of a protein is one of the major challenges in the era of genomics. With the rapid advancement in sequencing technologies and concerted genome projects, there is an increasing gap between the sequenced protein and functionally annotated proteins, (Casari et al., 1995; Yu et al., 2014; Agrawal et al., 2019d). Therefore, there is a requirement of automated computational methods that can identify the residues playing an essential role in protein functions. Protein–ligand interaction has been recognized as one of the important functions which play a vital function in all biological processes (Agrawal et al., 2019e). In the past, considerable efforts have been made to develop tools that can identify the ligand-interacting residues in a protein (Sousa et al., 2006). Initially, generalized methods have been developed which predicts the binding site or pockets in the proteins regardless of their ligand (Levitt and Banaszak, 1992; Laskowski, 1995; Hendlich et al., 1997; Dundas et al., 2006; Le Guilloux et al., 2009). Later on, it was realized that all ligands are not the same, and there is a wide variation in the shape and size of binding pockets. Therefore, researchers started developing ligand-specific methods (Chauhan et al., 2009; Chauhan et al., 2010; Chen et al., 2012; Yu et al., 2013a; Hu et al., 2016; Hu et al., 2018), and it was observed that these ligand-specific methods performed better than generalized methods (Chen et al., 2012; Yu et al., 2013b; Hu et al., 2016). Comprehensive information on the software developed for protein–small molecule interaction is reviewed in a paper by Agrawal et al. (2018).

All living organism consists of small molecular weight ligands or cofactors, which carries out an important function in some metabolic and regulatory pathways. S-adenosyl-L-methionine (SAM) is one such essential cofactor, first discovered in the year 1952. After ATP, SAM is the second most versatile and widely used small molecule (Cantoni, 1975). It is a natural substance present in the cells of the body and is a direct metabolite of L-methionine, which is an essential amino acid. SAM is a conjugate molecule of two ubiquitous biological compounds; (i) adenosine moiety of ATP and (ii) amino acid methionine (Catoni, 1953; Waddell et al., 2000). One of the most essential functions of the SAM is the transfer or donation of different chemical groups such as methyl (Wuosmaa and Hager, 1990; Thomas et al., 2004), aminopropyl (Lin, 2011), ribosyl (Kozbial and Mushegian, 2005), 5'deocxyadenosyl, and methylene group (Kozbial and Mushegian, 2005; Gana et al., 2013) for carrying out covalent modification of a variety of substrates. SAM is also used as a precursor molecule in the biosynthesis of nicotinamide phytosiderophores, plant hormone ethylene, spermine, and spermidine. It also carries out chemical reactions such as hydroxylation, fluorination which takes place in bacteria (Cadicamo et al., 2004). It has become the choice of various clinical studies and possess therapeutic value for treating diseases like osteoarthritis (Najm et al., 2004), cancer (Wagner et al., 2010; Chaib et al., 2011), epilepsy (Item et al., 2004), Alzheimer's (Borroni et al., 2004), dementia and depression (Bottiglieri et al., 1990; Rosenbaum et al., 1990), Parkinson (Zhu, 2004), and other psychiatric and neurological disorders (Bottiglieri, 1997). In the previous studies, it has been shown that mutation in the binding site of SAM has changed the protein function. For example, Aktas et al. showed that alanine substitution in the predicted SAM binding residues reduced the SAM binding affinity and enzyme activity dramatically (Aktas et al., 2011). Thus, there is a need to develop a method that can predict SAM binding sites in a protein sequence as it is an important ligand. Structure determination techniques (e.g., X-ray crystallography, Nuclear Magnetic Resonance (NMR), Cryo Electron Microscopy (Cryo-EM), Small Angle X-ray Scattering (SAXS) have been used to identify SAM interacting residue in protein. In addition, several experimental techniques have been used to investigate different aspects of protein–ligand interactions/protein–ligand binding affinity. Some of these widely used techniques are Isothermal Titration Calorimetry (ITC), Surface Plasmon Resonance (SPR), and Fluorescence Polarization (FP). Detailed description of these techniques and working principles has been provided in the article by Du et al. (2016). Although experimental techniques can elucidate ligand-interacting residue and thermodynamic profile for a given protein–ligand complex, these techniques are time-consuming, laborious, and expensive. Therefore, there is a requirement of highly robust and effective computational tools that can annotate the protein function using only its sequence. Also, *in silico* methods might provide new insights for the proteins whose 3D structure is not present in the literature.

## Materials and Methods

### Data Set Creation

Firstly, we extracted 244 SAM binding proteins Protein Data Bank (PDB) IDs from the PDB database whose structures are determined using X-ray crystallography. We considered only those proteins in which SAM was present as a free ligand, which resulted in 457 SAM binding protein chains. In the next step, we filtered all the sequences with a 40% sequence similarity using CD-HIT software (Huang et al., 2010) for creating a non-redundant data set. In previous studies, it has been shown that the performance of *in silico* method for protein annotation depends on the quality of protein structure used for its development (Chauhan et al., 2010; Patiyal et al., 2019). Thus, we remove all those structure from our data set whose resolution is poorer than 3Å. Finally, 145 protein chains have been obtained whose structure has been resolved at 3Å or better. Ligand Protein Contact (LPC) software (Sobolev et al., 1999) was used to extract the interatomic contact information of SAM interaction with residues present in the protein chains. LPC software implements surface complementarity theory to provide interatomic contacts in between ligand and residue. We used cutoff criteria of 4Å and called the residue SAM interacting if its contact with SAM is less than or equal to 4Å; else, the residue was assigned as SAM non-interacting. This criteria of data set creation is well-established and adopted in many previous studies for assigning ligand-interacting residues (Chauhan et al., 2010; Mishra and Raghava, 2010).

### Internal and External Validation

Data set was divided in a random manner into two parts: (i) training data set, which comprises 80% of the protein chains, and (ii) validation data set, which comprises remaining 20% of the protein chains. The training data set was used for training and testing the model using a fivefold cross-validation technique, which is called internal validation technique. In case of validation data set, it is only used for validating the performance of a model trained on training data set. This validation data set is also called independent or external validation data set as it is not used for training or testing of models. It is only used to validate the performance of best model trained on training data set.

Data sets were generated at the protein level instead of residue/pattern level as, in previous studies, it has been shown that data set created at pattern level is biased and shows higher performance (Yu et al., 2014). Data set was further classified into (i) balanced and (ii) realistic data set for model building and analysis studies. The balanced data set contains same number of SAM interacting and non-interacting residues (1,798 in training data set and 390 in the validation data set). The realistic data set consists of 1,798 SAM interacting residues and 33,314 SAM non-interacting residues in the training data set. In contrast, the validation data set consists of 390 SAM interacting residues and 6,715 SAM non-interacting residues. The internal data set was used for performing all kinds of analysis, i.e., composition, propensity, physiochemical properties, and statistical analysis.

### Fivefold Cross-Validation

The fivefold cross technique was performed for evaluating the performance of different prediction models in case of internal validation. In this process, data are divided into five equal parts, out of which four sets are used for model training, and the fifth is used for model testing. The process is repeated for five times during which each set is used for testing. Average performance obtained after five iterations is reported. This kind of performance evaluation has been used in many previous studies (Kumar et al., 2018; Nagpal et al., 2018). Once models were trained, their performance was tested on the validation data set, and this process is termed as external validation.

### Window or Pattern Size

We created overlapping patterns of each sequence of different window sizes ranging from 5-to 23-amino-acid length. If the pattern central residue is SAM interacting, it is designated as a positive pattern; otherwise, it was designated as a negative pattern. (L−1)/2 (where L is pattern length) number of “X,” a dummy residue was added at both the termini of the protein chain for generating patterns for terminus residues.

### Binary Profile

We generated binary profile of each pattern by assigning binary values to the amino acids in fixed length pattern. A vector of dimension 21 represented each amino acid present in the pattern hence leading to final vector of N × 21, where N is pattern length. For example, residue “A” was represented by [1,0,0,0,0,0,0,0,0,0,0,0,0,0,0,0,0,0,0,0,0]; which contains 20 amino acids and one dummy amino acid “X.” X was represented by [0,0,0,0,0,0,0,0,0,0,0,0,0,0,0,0,0,0,0,0,0] (Agrawal and Raghava, 2018; Agrawal et al., 2019b).

### Position-Specific Scoring Matrix

Position-specific scoring matrix (PSSM) profiles containing evolutionary information have been shown as an important feature in many previous studies for predicting protein–ligand interaction and other bioinformatics problems (Chen et al., 2012; Yu et al., 2013a). PSSM profiles of a sequence were generated using Position-Specific Iterative Basic Local Alignment Search Tool (PSI-BLAST) and searching against the Swiss Prot database. In total, three iterations were performed with an E-value cutoff of 0.001 against each sequence. The original PSSM profiles obtained were further normalized to get value in between 0 and 1, followed by calculation of the position-specific score for each residue. The final matrix obtained consists of 21 × N elements (20 amino acids residue and one dummy residue “X”). Here N is the length of the pattern.

### Machine Learning Techniques

We implemented the Python-based machine learning package SCIKIT-learn (Pedregosa et al., 2011) for developing prediction models. We implemented the support vector classifier (SVC), random forest classifier (RF), ExtraTree classifier (ETree), K-nearest neighbor (KNN), multilayer perceptron (MLP), and Ridge classifier for developing prediction models. We optimized different parameters on our internal data set using the Grid Search parameter present in the package before model prediction development.

### Statistical Study

P-value was computed to observe the statistical significance between the composition and propensity values of SAM interacting and non-interacting residues. The significance level considered for computing P-value was 0.05.

### Evaluation Parameters

Performance of developed prediction models was evaluated in terms of sensitivity (Sen), specificity (Spc), accuracy (Acc), Matthews Correlation Coefficient (MCC), and area under receiver operating characteristics (AUROC) as shown in previous studies (Le and Ou, 2016a; Le and Ou, 2016b; Le et al., 2017). “pROC package” implemented in R was used for computing AUROC (Title Display and Analyze ROC Curves, 2019). The formula for calculating is explained in Equations 1–4.

1 $$\mathrm{Sensitivity}=\frac{\mathrm{TP}}{\mathrm{TP}+\mathrm{FN}}\times 100$$

2 $$\mathrm{Specificity}=\frac{\mathrm{TN}}{\mathrm{TN}+\mathrm{FP}}\times 100$$

3 $$\mathrm{Accuracy}=\frac{\mathrm{TP}+\mathrm{TN}}{\mathrm{TP}+\mathrm{FP}+\mathrm{TN}+\mathrm{FN}}\times 100$$

4 $$\mathrm{MCC}=\frac{\left(\mathrm{TP}\times \mathrm{TN}\right)-\left(\mathrm{FP}\times \mathrm{FN}\right)}{\sqrt{\left(\mathrm{TP}+\mathrm{FP}\right)\left(\mathrm{TP}+\mathrm{FN}\right)\left(\mathrm{TN}+\mathrm{FP}\right)\left(\mathrm{TN}+\mathrm{FN}\right)}}$$

where TP is correctly predicted positive value, TN is the correctly predicted negative value, FP is the actual negative value which has been wrongly predicted as positive, and FN is the positive value which has been wrongly predicted as negative.

## Results

### Analysis of SAM Binding Sites

#### Composition Analysis

**Figure 1:**
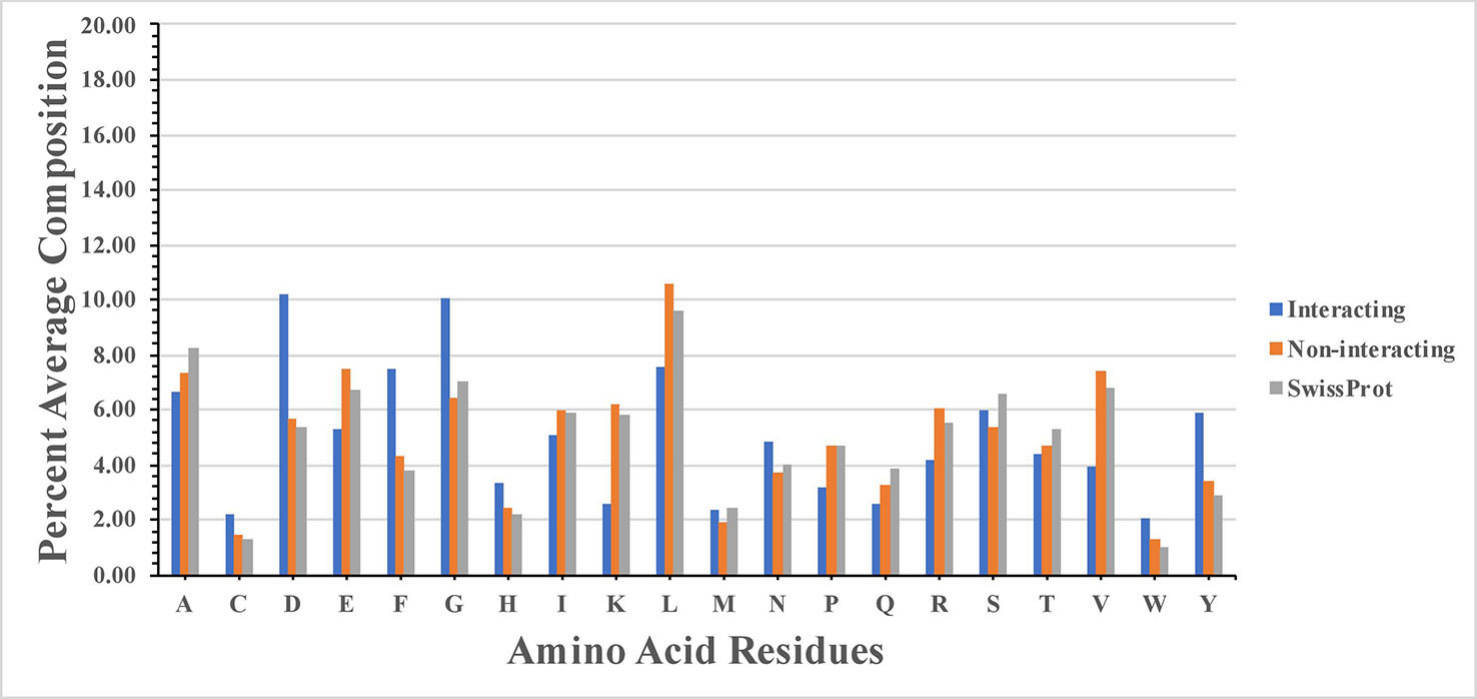
Percentage composition of S-adenosyl-L-methionine (SAM) interacting and non-interacting residues.

High frequency of residues C, D, F, G, H, N, W, and Y was observed in SAM interacting sites (**Figure 1**). Our study agrees with the previous study (Gana et al., 2013), where authors show that SAM interacting proteins belong to fold type I families where charged and small amino acids (D, E, K, H, Y, and G) are involved in the interaction. The study showed that SAM interactions were predominantly stabilized by H-bonds and the atoms of SAM responsible for interaction with protein. These atoms are N, N1, and N6 sites of the adenine ring, O2* and O3* sites of sugar moiety, and terminal N, O, and OXT. Amino acids interacted at N1 site included mostly hydrophobic residues L, V, A, C, F, M, and G. Residues interacted at N6 site included polar residues Q and S, along with charged residues D and E. Statistical analysis showed that there is statistical significance in the composition of SAM interacting and non-interacting residues which include C, D, E, F, G, H, K, L, N, P, R, V, W, and Y. P-value computed for the SAM interacting and the non-interacting residue is provided in the **Supplementary Table S1**.

#### Normalized Propensity Analysis

We also analyzed the normalized propensities of amino acid residues in SAM interacting and non-interacting sites. We observed that propensities of residues like C, D, F, G, H, M, N, S, W, and Y were higher in SAM interacting sites (**Supplementary Figure S1**). High statistical significance in the propensity value of SAM interacting and the non-interacting residue was observed with P-value 0.0 for all residues.

#### Physiochemical Properties Analysis

We found that SAM interacting sites are rich in acidic, small, polar, and aromatic amino acids, as shown in **Supplementary Figure S2**. Our study agrees with the previous studies (Gana et al., 2013; Nagarajan et al., 2015).

### Machine Learning Model Performance Using Binary Patterns

**Figure 2:**
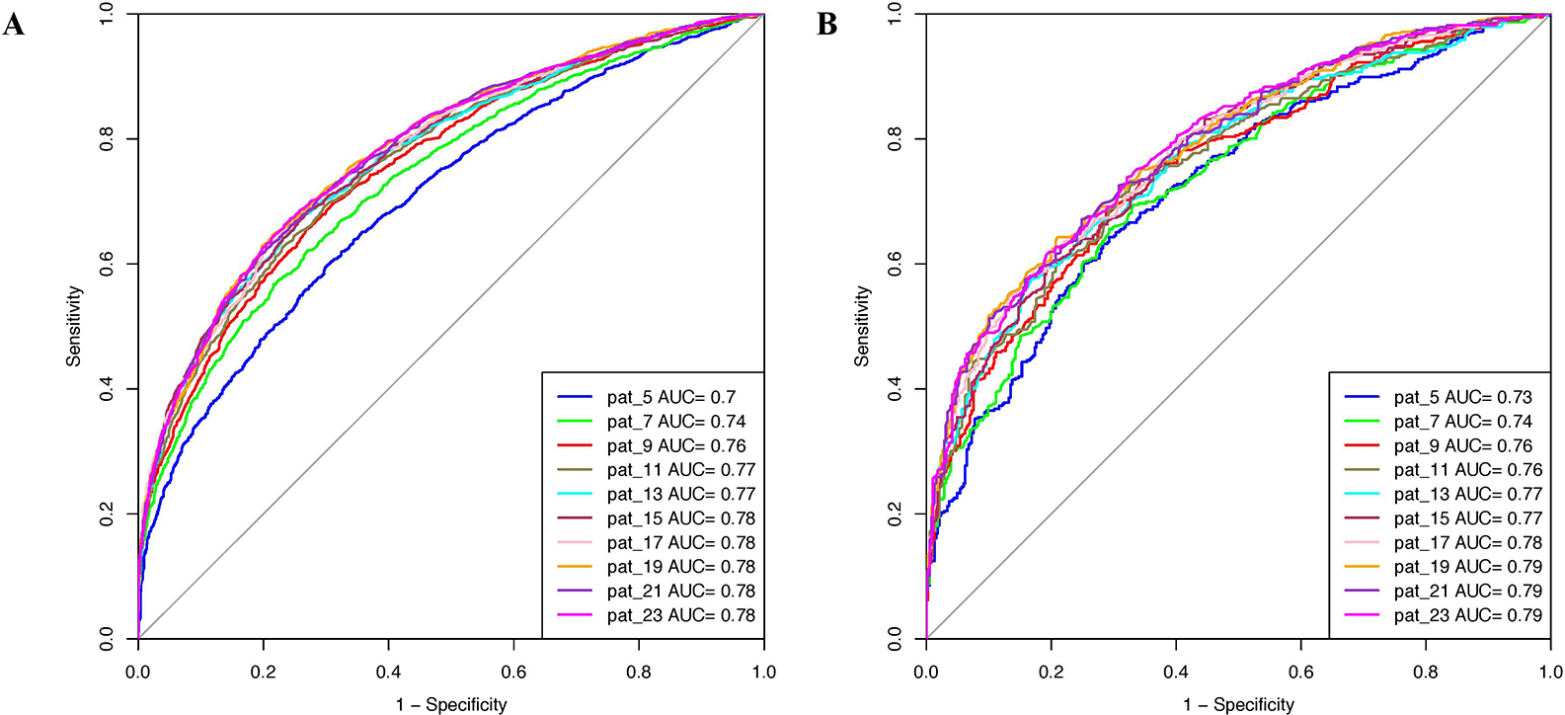
Area under receiver operating characteristics (AUROC) plots obtained for various window length developed using binary profile on balanced data set for **(A)** training data set and **(B)** validation data set.

Various machine learning models were developed using binary patterns for window size 5–23 on the balanced data set. We compiled the result obtained using RF classifier for each window size in **Table 1** as this classifier performed best for most of the patterns. AUROC plot obtained for both training and validation data sets is shown in **Figures 2A, B**, respectively. In our analysis, we observed that RF based prediction model performed best among all the prediction models for the window size 21. The model achieved an accuracy of 70.79%, 0.42 MCC, and 0.78 AUROC on the training data set and accuracy of 70.85%, 0.42 MCC, and 0.79 AUROC on the validation data set. Detail result obtained for each window size by different machine learning techniques is provided in **Supplementary Tables S2–S11**.

**Table 1:** The performance of random forest model developed using amino acid sequence (binary pattern) for individual window size on balanced data set.

Pattern size	Training data set					Validation data set				
	Sen	Spc	Acc	MCC	AUROC	Sen	Spc	Acc	MCC	AUROC
Pat5	65.71	61.67	63.69	0.27	0.70	66.06	63.21	64.64	0.29	0.71
Pat7	69.87	64.31	67.09	0.34	0.74	69.43	66.58	68.01	0.36	0.74
Pat9	72.05	65.66	68.86	0.38	0.76	69.95	68.65	69.30	0.39	0.76
Pat11	69.19	70.26	69.73	0.39	0.77	65.03	71.76	68.39	0.37	0.76
Pat13	73.06	66.05	69.56	0.39	0.77	70.98	65.03	68.01	0.36	0.77
Pat15	69.58	70.71	70.15	0.40	0.78	66.06	72.02	69.04	0.38	0.78
Pat17	70.37	71.10	70.74	0.41	0.78	67.36	71.50	69.43	0.39	0.78
Pat19	70.54	71.32	70.93	0.42	0.78	67.36	73.83	70.60	0.41	0.79
Pat21	70.37	71.21	70.79	0.42	0.78	67.62	74.09	70.85	0.42	0.79
Pat23	70.76	70.99	70.88	0.42	0.78	68.39	71.76	70.08	0.40	0.79

Sen, sensitivity; Spc, specificity; Acc, accuracy; MCC, Matthews Correlation Coefficient; AUROC, area under the receiver operating characteristic curve.

### Machine Learning Model Performance Using Evolutionary Information (PSSM Profile)

**Figure 3:**
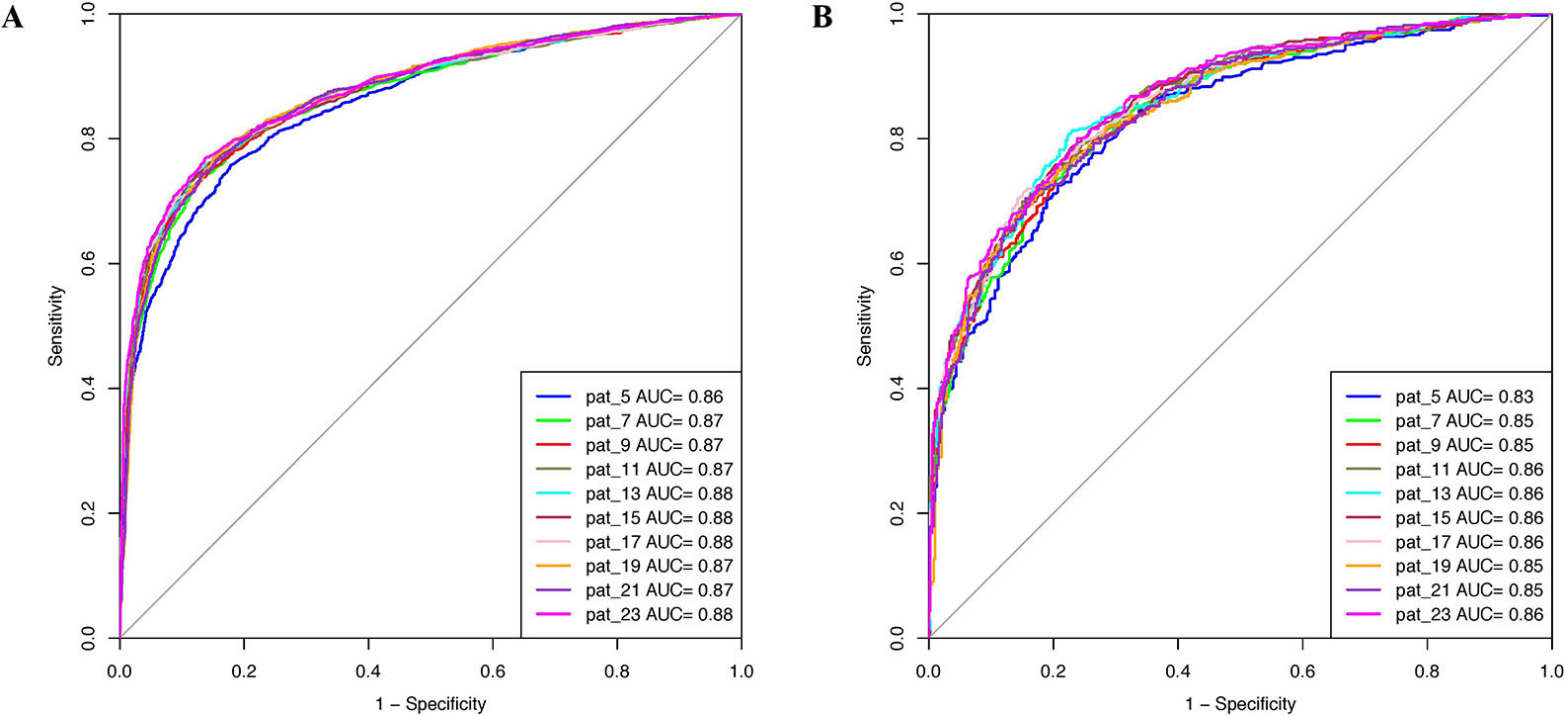
AUROC plots obtained for various window length developed using evolutionary profile on balanced data set for **(A)** training data set and **(B)** validation data set.

Prediction models were developed using PSSM profiles for all the considered window size on the balanced data set. The result obtained using ETree classifier for each window size is compiled in **Table 2** as this classifier performed best for most of the patterns. AUROC was plotted for the training (**Figure 3A**) and validation data set (**Figure 3B**). We observed that, in the case of PSSM profiles, the performance of the prediction models were increased. ETree classifier model developed on the window size 17 performed best among all the developed models. It achieved the highest accuracy of 80.39%, MCC of 0.61, and AUROC of 0.88 on the training data set, whereas, on the validation data set, it achieved accuracy of 77.07%, MCC of 0.54, and AUROC of 0.86. The result obtained by different classifiers on each window size has been provided in the **Supplementary Tables S12–S21**.

**Table 2:** The performance of ExtraTree classifier model developed using PSSM profile for individual window size on balanced data set.

Pattern size (classifier)	Training data set					Validation data set				
	Sen	Spc	Acc	MCC	AUROC	Sen	Spc	Acc	MCC	AUROC
Pat5	79.18	75.76	77.47	0.55	0.86	73.83	76.17	75.00	0.50	0.83
Pat7	79.85	79.12	79.49	0.59	0.87	73.32	79.53	76.42	0.53	0.85
Pat9	81.48	76.49	78.98	0.58	0.87	76.68	77.46	77.07	0.54	0.85
Pat11	81.54	76.32	78.93	0.58	0.87	75.39	78.50	76.94	0.54	0.86
Pat13	79.29	80.42	79.85	0.60	0.88	74.87	81.61	78.24	0.57	0.86
Pat15	81.59	77.10	79.35	0.59	0.88	76.68	78.24	77.46	0.55	0.86
Pat17	79.24	81.54	80.39	0.61	0.88	72.54	81.61	77.07	0.54	0.86
Pat19	82.21	76.04	79.12	0.58	0.88	76.68	78.24	77.46	0.55	0.86
Pat21	79.85	81.59	80.72	0.61	0.88	74.87	81.87	78.37	0.57	0.86
Pat23	79.91	81.14	80.53	0.61	0.88	73.06	81.61	77.33	0.55	0.86

PSSM, position-specific scoring matrix.

### Machine Learning Model Performance Using Hybrid Feature

We also developed models on the hybrid feature where we sum up the values of binary profile and the evolutionary information obtained for the residue. The result obtained for each window length using SVC is compiled in **Supplementary Table S22** as it performed best for maximum patterns. AUROC plot for the training data set and validation data set is provided in **Supplementary Figures S3A** and **B**, respectively. SVC obtained a maximum accuracy of 80.58%, MCC of 0.61, and AUROC of 0.89 on the training data set for window size 19. In the case of the validation data set, the accuracy of 78.50%, MCC of 0.57, and AUROC of 0.87 were obtained. The result for all the window sizes obtained by different classifiers is provided in **Supplementary Tables S23–S32**.

### Machine Learning Models Performance on the Realistic Data Set

**Figure 4:**
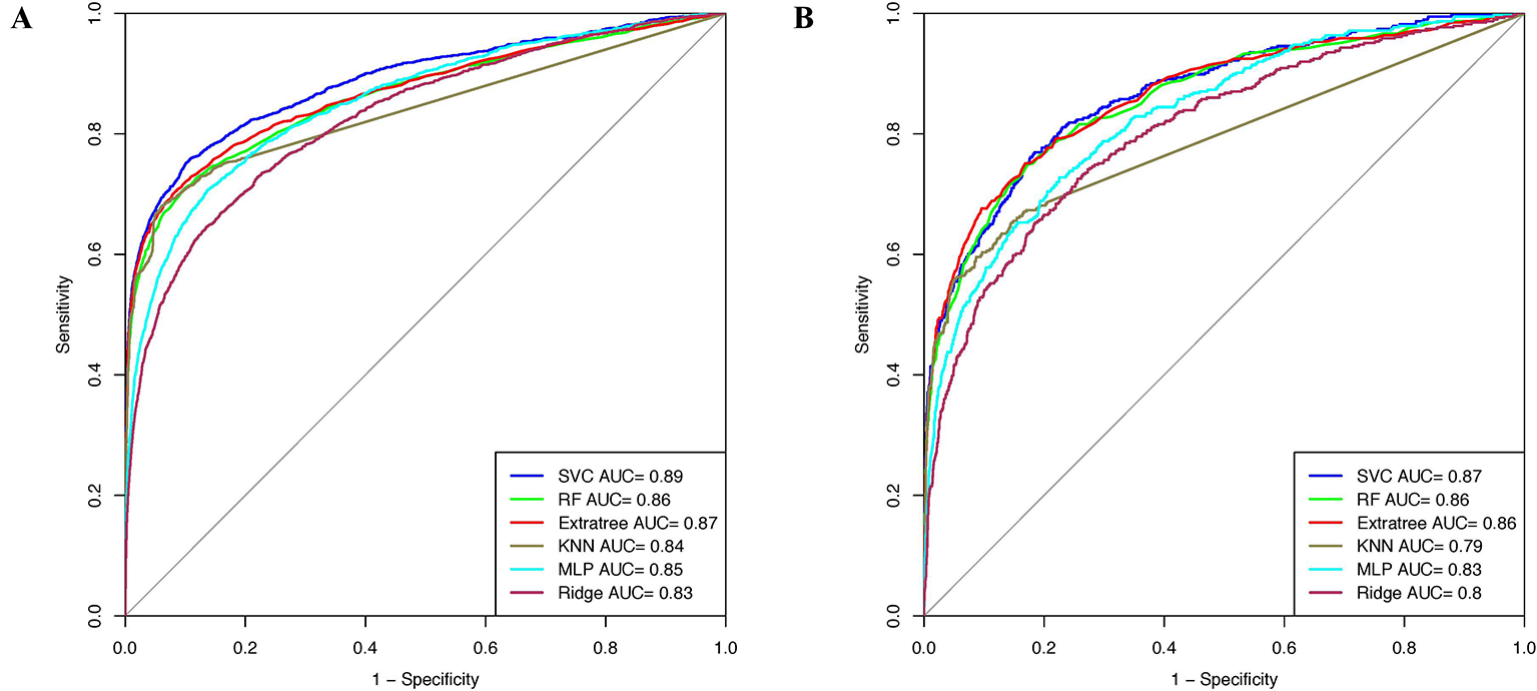
AUROC plots obtained for window length 17 developed using evolutionary profile on realistic data set for **(A)** training data set and **(B)** validation data set.

Window size 17 was found to be the optimum window size as the model developed using the PSSM profile performed best among all the models. Therefore, we used this window size for developing prediction models on the realistic data set using the PSSM profile as an input feature. When balanced specificity and sensitivity were considered, SVC based model achieved maximum MCC value of 0.32 on the training data set and 0.31 on the validation data set. However, MCC value increases to 0.61 on the training data set and 0.52 on the validation data set when balanced sensitivity and specificity were not taken into account (**Table 3**). The AUROC achieved on the training data set and validation data set was 0.89 and 0.87, respectively (**Figure 4**).

**Table 3:** The performance of PSSM profile based models developed using different machine learning techniques for window size 17 on realistic data set.

Machine Learning Techniques	Main Data Set					Validation Data Set				
	Sen	Spc	Acc	MCC	AUROC	Sen	Spc	Acc	MCC	AUROC
SVC^#^	53.65	98.94	96.64	0.61	0.89	36.53	99.40	95.99	0.52	0.87
SVC^*^	81.48	80.14	80.21	0.32	0.89	77.72	79.88	79.76	0.31	0.87
RF^#^	43.27	99.44	96.59	0.58	0.86	36.53	98.97	95.58	0.48	0.86
RF^*^	80.25	74.64	74.93	0.27	0.86	76.94	79.73	79.58	0.30	0.86
ETree^#^	46.13	99.42	96.72	0.60	0.87	47.67	97.93	95.20	0.50	0.86
ETree^*^	78.90	79.79	79.74	0.31	0.87	74.09	83.70	83.18	0.33	0.86
KNN^#^	46.91	99.27	96.61	0.59	0.84	34.72	99.36	95.85	0.50	0.79
KNN^*^	76.04	79.72	79.53	0.29	0.84	68.13	80.56	79.89	0.27	0.79
MLP^#^	37.49	98.59	95.49	0.45	0.85	34.46	98.01	94.55	0.39	0.83
MLP^*^	78.84	75.84	76.00	0.27	0.85	65.28	83.26	82.28	0.28	0.83
Ridge^#^	38.61	97.55	94.55	0.39	0.83	37.31	96.13	92.93	0.33	0.80
Ridge^*^	79.57	67.37	67.99	0.22	0.83	77.46	67.42	67.97	0.21	0.80

*Balanced sensitivity and specificity, ^#^maximum MCC, SVC, support vector classifier; RF, random forest; ETree, ExtraTree; KNN, K-nearest neighbors; MLP, multilayer perceptron.

### Implementation of Model in Web Server

In order to help biologists predict SAM interacting residues, we implemented our best models in a web server, SAMbinder. The web server consists of several modules such as “Sequence,” “PSSM Profile,” “Peptide Mapping,” “Standalone,” and “Download.” These modules have been explained below in detail.

**(i) Sequence:** This module allows users to predict SAM interacting residue in a protein from its primary sequence. A user can submit either single or multiple sequences or upload the sequence file in the FASTA format and can select the desired probability cutoff and machine learning classifier for prediction. The module utilizes the binary profile as an input feature, and several machine learning models have been implemented into it. The classifier provides the prediction score, which is normalized in between propensity score 0–9. Residues having the propensity score equal or above the selected cutoff threshold are highlighted in blue color, and remaining residues are highlighted in black color. Blue color indicates that the probability of these residues in SAM binding is high in comparison to the residues present in black color. The result is downloadable in the “csv” file format and will be sent to email also if the user has provided the email.

**(ii) PSSM profile:** As the name suggests, this module utilizes the PSSM profile as an input feature for predicting SAM interacting residues in a given protein sequence. This feature is better than the binary profile; however, the only limitation is that it is very computer-intensive. Therefore, a user can use this module if the number of sequences is very few. The output is provided in the same format as the Sequence module provides. For predicting multiple sequences using the PSSM profile, we suggest for the user to utilize the standalone version of the software.

**(iii) Peptide mapping:** In this module, we have provided the facility where a user can map the peptide that contains SAM interacting central residue. We pre-computed propensity (between 0 and 9) of each tri and pentapeptide, which contains SAM interacting central residues from known PDB protein structure. The propensity was computed using all SAM interacting protein chains, i.e., redundancy was not removed to avoid loss of information. Once a user submits sequence in FASTA format, all the possible segments of selected length are generated and mapped on the protein sequence along with the propensity score. Based on that mapping server predicts whether the peptide segment is SAM interacting or non-interacting. If the propensity of residue is equal to greater than the selected threshold, it is known as SAM interacting residue.

### Standalone

Standalone of SAMbinder is Python-based and is available at the GitHub site. The user can download it from the website https://github.com/raghavagps/sambinder/. SAMbinder standalone version is also implemented in the docker technology. Complete usage of downloading the image and its implementation is provided in the docker manual “GPSRdocker” (Agrawal et al., 2019a) which can be downloaded for the website https://webs.iiitd.edu.in/gpsrdocker/.

## Discussion

SAM is an essential metabolic cofactor/intermediate, which is found in almost every cellular life form and enzyme. It is a sulfonium molecule and hybrid of two structural molecules methionine and adenosine. The primary function of SAM is to perform as a methyl donor to N-, C-, O-, or S- nucleophiles under the catalysis of enzymes known as SAM-dependent methyltransferases (MTases). The reaction is carried out through the SN2 type mechanism, where the nucleophilic attack takes place at the methyl group adjacent to the sulfonium center. The reaction mentioned above leads to the transmethylation of various biomolecules (DNA, RNA, proteins, carbohydrates, and other small metabolites). These biomolecules are involved in significant biochemical mechanisms such as cellular signaling, epigenetic regulation, and metabolite degradation. Hence, this transmethylation reaction is of broad biological significance (Zhang and Zheng, 2016).

**Figure 5:**
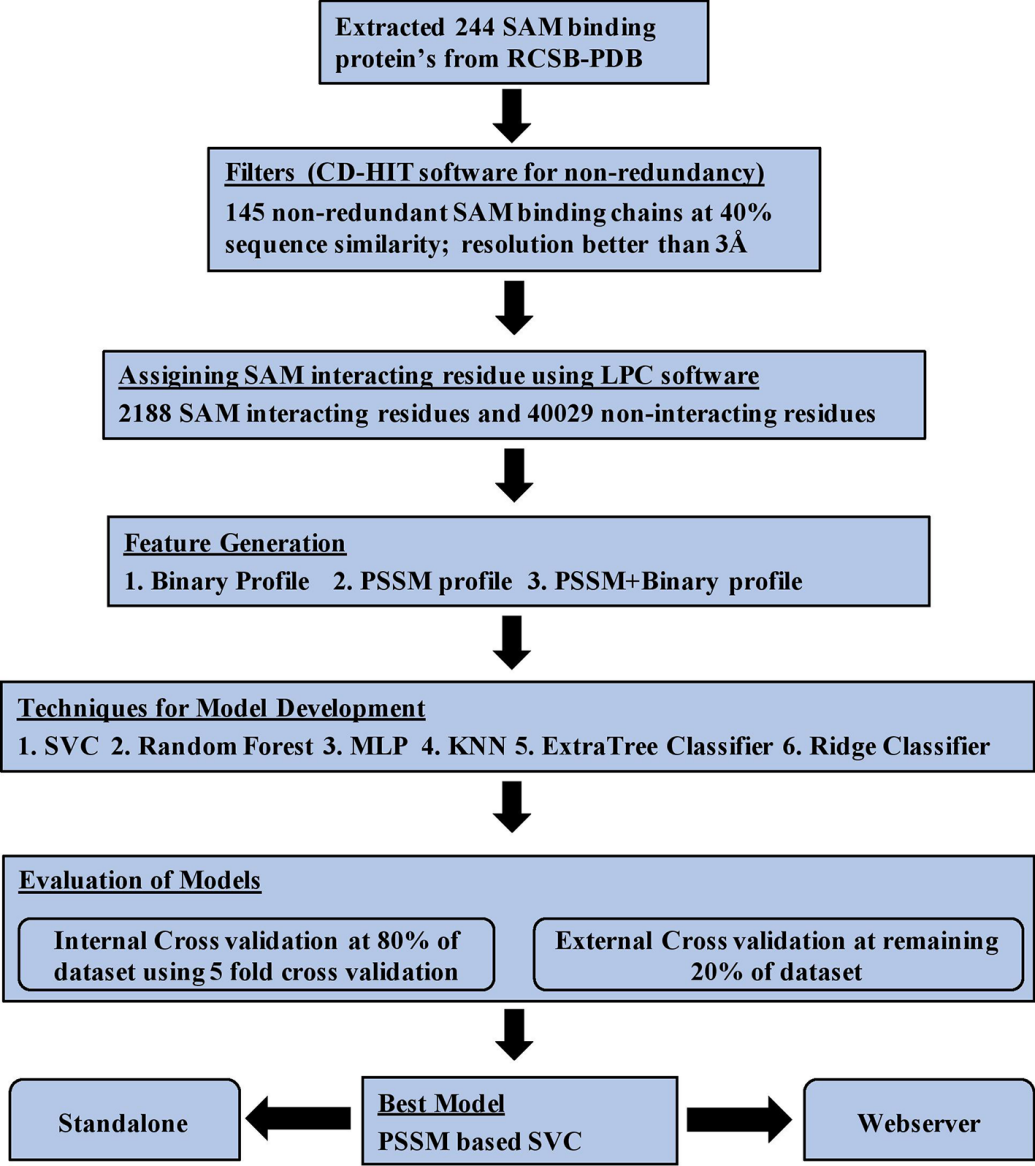
Architecture of SAMbinder.

SAM binding proteins are predominant in two major types of folds: (i) Rossman fold and Triosephosphate Isomerase (TIM) barrel fold and different motifs (Motif I–VI) (Gana et al., 2013). SAM binding proteins play a vital role in many metabolic and regulatory pathways in almost all forms of the living organism and acts as a potential drug target in several diseases. In Europe, SAMe is used as a drug for treating diseases like liver disorder, depression, fibromyalgia, and osteoarthritis. It has also been used as dietary supplements in the United States for supporting the bones and joints. Therefore, it is very important to predict the SAM interacting residues in a given protein. We analyzed various properties of SAM interacting protein chains such as composition, propensity, and physiochemical properties and developed various machine learning models for predicting SAM interacting residue in new protein using a number of input features. The models were first developed on the balanced data set and different window sizes. We observed that the model developed using the PSSM profile and window size 17 performed best among all the models. The performance of the models was also validated on an independent data set and an additional data set. Python-based machine learning package scikit-learn was implemented for developing the prediction models. In order to assist the scientific community, we have created a Python-based standalone version of our software and also developed a web server where a user can predict the SAM interacting residues in the target protein. The server can be freely accessible at http://webs.iiitd.edu.in/raghava/sambinder. The complete workflow of SAMbinder is shown in **Figure 5**.

## Data Availability Statement

The raw data supporting the conclusions of this manuscript will be made available by the authors, without undue reservation, to any qualified researcher.

## Author Contributions

PA collected and compiled the data sets. PA performed the experiments. PA and GR developed the web interface. PA and GM developed the standalone software. PA and GR analyzed the data and prepared the manuscript. GR conceived the idea and coordinated the project. All authors read and approved the final paper.

## Funding

This work was supported by the J.C. Bose National Fellowship, Department of Science and Technology, government of India.

## Conflict of Interest

The authors declare that the research was conducted in the absence of any commercial or financial relationships that could be construed as a potential conflict of interest.

The reviewer MG declared a past co-authorship with one of the authors GR to the handling editor.
